# The Burden of Ventricular Premature Complex Is Associated With Cardiovascular Mortality

**DOI:** 10.3389/fcvm.2021.797976

**Published:** 2022-02-03

**Authors:** Po-Tseng Lee, Ting-Chun Huang, Mu-Hsiang Huang, Ling-Wei Hsu, Pei-Fang Su, Yen-Wen Liu, Meng-Hsuan Hung, Ping-Yen Liu

**Affiliations:** ^1^Institute of Clinical Medicine, College of Medicine, National Cheng Kung University, Tainan, Taiwan; ^2^Division of Cardiology, Department of Internal Medicine, National Cheng Kung University Hospital, College of Medicine, National Cheng Kung University, Tainan, Taiwan; ^3^Department of Statistics, College of Management, National Cheng Kung University, Tainan, Taiwan

**Keywords:** ventricular premature complex (VPC), 24-h Holter electrocardiogram, cardiovascular mortality, cox regression analysis, Fine and Gray's model

## Abstract

**Background:**

Ventricular premature complex (VPC) is one of the most common ventricular arrhythmias. The presence of VPC is associated with an increased risk of heart failure (HF).

**Method:**

We designed a single-center, retrospective, and large population-based cohort to clarify the role of VPC burden in long-term prognosis in Taiwan. We analyzed the database from the National Cheng Kung University Hospital-Electronic Medical Record (NCKUH-EMR) and NCKUH-Holter (NCKUH-Holter). A total of 19,527 patients who underwent 24-h Holter ECG monitoring due to palpitation, syncope, and clinical suspicion of arrhythmias were enrolled in this study.

**Results:**

The clinical outcome of interests involved 5.65% noncardiovascular death and 1.53% cardiovascular-specific deaths between 2011 and 2018. Multivariate Cox regression analysis, Fine and Gray's competing risk model, and propensity score matching demonstrated that both moderate (1,000–10,000/day) and high (>10,000/day) VPC burdens contributed to cardiovascular death in comparison with a low VPC burden (<1,000/day).

**Conclusion:**

A higher VPC burden *via* Holter ECG is an independent risk factor of cardiovascular mortality.

## Introduction

Ventricular premature complex (VPC) is one of the most common ventricular arrhythmias and is strongly associated with heart failure (HF), all-cause hospitalization, and cardiovascular hospitalization (1–3). A high VPC burden (>10,000/day) can reduce systolic blood pressure and is reportedly detrimental to the left ventricular systolic function (2, 4, 5). The accumulated data demonstrate that the elimination of VPC by either catheter ablation or antiarrhythmic drugs (AADs) not only relieves symptoms but also improves the left ventricular systolic function in patients with preexisting left ventricular systolic dysfunction or VPC-induced cardiomyopathy (6–10). Of interest, cardiac mortality, transplantation, and hospitalization are reduced in patients with frequent VPC after receiving a successful catheter ablation (11).

Regarding the overall effect of VPC burden on the cardiovascular system, several limitations exist in the current research literature. First, the size of previous cohorts using 24-h Holter monitoring to evaluate daily VPC burden is limited that the largest population size reported to date is 5,778 patients in a single cohort (12). Although the other all-comer studies and meta-analysis contained a large population size, the lack of daily VPC counts creates difficulty in evaluating the relationship between daily burden and prognosis (13). Second, several significant clinical comorbidities could be confounding factors for all-cause death and cardiovascular death. These factors include age, coexisting diseases like diabetes mellitus (DM), chronic kidney disease (CKD), and stroke, the presence of ventricular tachycardia, and HF. Third, although the presence of exercise-induced VPC was reported to be associated with cardiovascular death, the effect of VPC burden on cardiovascular death has not been directly reported until a recent subgroup analysis from the CHF-STAT study demonstrated that amiodarone could suppress PVC burden and improve survival (10, 13, 14). To consolidate the role of VPC burden on patient prognosis, particularly cardiovascular death, we designed a single-center, retrospective study with a large population-based cohort.

## Original Research

### Materials and Methods

#### Data Source and Study Population

This retrospective, observational study utilized the National Cheng Kung University Hospital-Electronic Medical Record (NCKUH-EMR) database and the NCKUH-Holter (NCKUH-Holter) database. The methodology for establishing these databases has been described and validated in our previous studies (15–17). The study population included all patients who underwent 24-h Holter ECG monitoring between July 1, 2011, and December 31, 2018, at the NCKUH. An indication for Holter ECG monitoring included clinical suspicion of arrhythmia, syncope, and palpitation. With regard to patients who underwent multiple Holter studies, the index Holter study was defined as that with the largest VPC burden. We excluded patients with an incomplete chart review, patients under 18 years of age, patients who had a loss of follow-up or were not regularly followed for over 6 months, and patients who did not complete a Holter study. The clinical data used in this study were obtained from NCKUH with IRB approval (B-ER-108-290), and it was registered to ClinicalTrials.gov (http://clinicaltrials.gov/ct2/show/NCT03877614).

#### Clinical Characteristics and Outcome

Data were collected from the NCKUH-EMR database. This database was excavated from the electrical medical record system of the NCKUH using a data mining technique. Clinical diagnosis from the database was validated by a random sample survey performed by three cardiologists. The accuracy of the sample survey was 99%. Clinical characteristics of interest included age, gender, and history of diseases, including DM, dyslipidemia, hypertension, stroke, CKD, HF, and cardiovascular disease (CVD). HF was defined as a left ventricular ejection fraction of <50%, and CVD was defined as composite diseases, including coronary artery disease, stroke, and peripheral artery disease. The clinical outcomes of interest in this study were all-cause mortality and cardiovascular death. Mortality data were retrieved from the medical records of our hospital.

#### Ascertainment of Death and Definition of Cardiovascular Death

The ascertainment of death was performed by data mining and a chart review. As this is a retrospective cohort study enrolling patients with a regular follow-up from 2011 to 2018, the validation of death could be achieved by a chart review. A telephone interview was used for uncertain patients to validate the survival status. The primary cause of death was defined as the underlying disease or injury that initiated the course of events that resulted in death. Cardiovascular death was defined as death attributable to acute myocardial infarction, sudden cardiac death, HF, stroke, cardiovascular procedures, cardiovascular hemorrhage, and death due to any other cardiovascular cause (18). The cause of death for each patient was determined by comprehensive judgment according to the certification of death and discharge diagnosis recorded in the EMR, which was reviewed by a cardiologist (P-TL) and validated by the two other independent cardiologists (T-CH and M-HH).

#### 24-H Holter ECG Variable

Data were collected from the NCKUH-Holter database as formal Holter reports are contained in this database. VPC was identified by a simultaneous 3-channel 24-h Holter monitoring (NorthEast Monitoring, Inc., DR200/HE Digital Recorder). Holter monitor raw data were edited by the four experienced technicians (Ms. Y.L.Chen, Ms. S.F. Hsu, Ms. C.Y. Tseng, and Ms. Y.S. Hsueh), and all arrhythmic episodes, unknown strips, and final formal Holter reports were reviewed and confirmed by five senior cardiologists. Variables of interest from the Holter data in this study included VPC burden, atrial premature complex (APC) burden, supraventricular tachycardia (SVT), and ventricular tachycardia (VT). Both monomorphic and polymorphic VPCs were included in the study. Incomplete or uninterpretable Holter studies due to the dislodgement of the electrogram patch, machine malfunction, uncooperative patients, or standstill strips were excluded. All valid Holter studies were eligible for further analysis. A pilot validation study was performed to guarantee the accuracy of the database. A total of 200 patients were randomly selected, and four random traits were obtained, such as baseline characteristics, comorbidities, or medications. Each patient was manually reviewed by two of the three cardiovascular physicians (P-TL, T-CH, and M-HH). The accuracy rate of all traits was 99.12% (793/800).

### Statistical Analysis

Baseline clinical characteristics were reported as means ± SDs for continuous variables, median [interquartile range (IQR)] for non-normal distribution variables, and as percentages for categorical variables. As the daily VPC burden is not a natural distribution, a natural log transformed VPC was created as a representative continuous variable. Student's *t*-test and Chi-squared test with Yates' correction was used to analyze the continuous and categorical variables, respectively. A univariate and multivariate Cox regression model was performed to analyze the contribution of clinical and Holter variables to cardiovascular death. The adjusted variables included age, gender, DM, dyslipidemia, hypertension, CKD, HF, CVD, antiplatelet drugs, AADs, beta blocker, and VT. A propensity score matching method was used to analyze the clinical outcomes (all-cause death and cardiovascular death) of patients with low (<1,000/day), moderate (1,000–10,000/day), and high (>10,000) VPC burdens. Crude event rates from Kaplan–Meier survival curves were compared between these groups using the log-rank test. To further elucidate the contribution of VPC burden to cardiovascular death, cumulative incidence functions (CIFs) were used to estimate the incidence of each of the different types of competing risks. A Fine–Gray subdistribution hazard model was used to estimate the prognosis of VPC in the presence of other competing risks (19).

## Result

### Baseline Characteristics, VPC Burden, and Clinical Outcome of the Study Cohort

**Figure 1:**
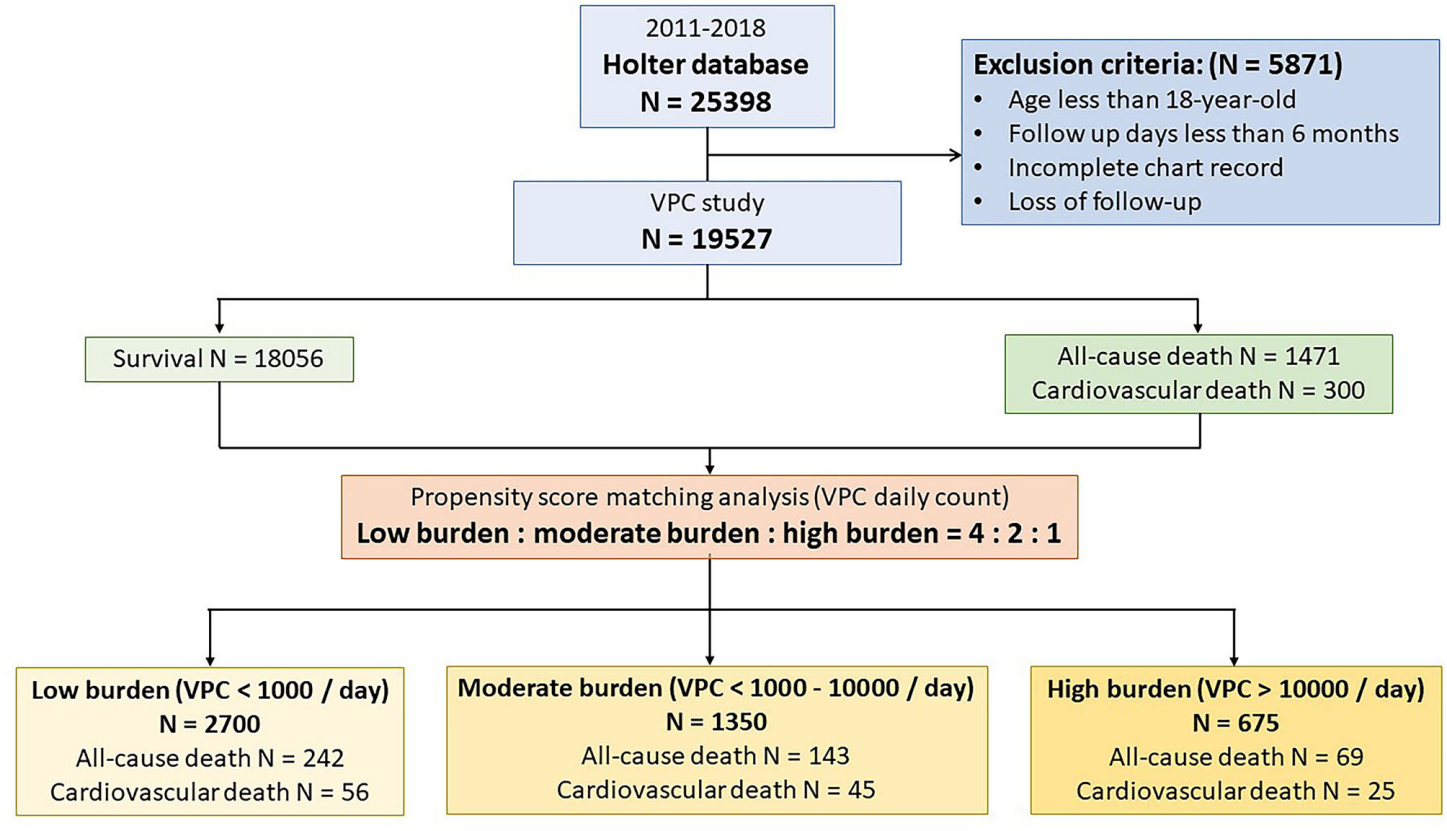
Flow chart of the study protocol. A total of 19,527 patients were enrolled in the study. During the follow-up period, 1,471 patients suffered from death. Cardiovascular death accounts for 20.4% (*n* = 300) of all-cause death. A propensity score matching analysis was performed to analyze the effect of ventricular premature complex (VPC) burden on all-cause death and cardiovascular death.

From 2011 to 2018, we consecutively analyzed 30,488 records of Holter monitoring from 25,398 patients. Patients with an incomplete chart review and who aged less than 18 years and had a loss of follow-up or were followed up for less than 6 months were excluded from this study. About 24,071 Holter monitoring records belonging to 19,527 patients were analyzed. The study design is illustrated in Figure 1. The study population was predominately 52.8% male with a mean age of 59.6 ± 17.5 years, 22.4% DM, 46.0% dyslipidemia, 49.3% hypertension, 8.7% experienced a stroke, 19.8% CKD, 12.4% HF, and 22.9% CVD. In terms of the Holter study, the mean VPC burden was 1,129 ± 4,422/day, and 6.3% of the study patients had VT. The VPC count was translated to logarithm due to the right skew distribution. The mean log VPC was 3.0 ± 2.9. Non-sustained VT was detected in 6.3% of the study population. During a mean 955-day follow-up, 1,471 (7.53%) patients died. Among the deceased patients, 300 (1.53%) deaths were attributed to cardiovascular death, 1,103 (5.65%) deaths were attributed to non-cardiovascular death, and the remaining 71 deaths were of an unknown etiology due to death occurring prior to arriving at the hospital or an incomplete medical record.

### VPC Burden Is Associated With All-Cause and Cardiovascular Death

To evaluate whether VPC burden is associated with all-cause death or cardiovascular death, the study cohort was divided into three groups based on the VPC counts, including a low VPC burden defined as <1,000/day (*n* = 16,996), a moderate VPC burden defined as 1,000–10,000/day (*n* = 1,856), and a high VPC burden defined as >10,000/day (*n* = 675) based on the previous literature (2). The median and IQR of daily VPC count in the three groups was 5 (1–44), 2,712 (1,618–4,982), and 17,726 (13,306–25,704). Meanwhile, the median and IQR of daily VPC burden in the three groups was <0.1% (<0.1– <0.1%), 2.6% (1.5–4.9%), and 17.5% (13.1–25.4%), respectively. In comparison to the low burden group, the moderate and high burden groups had more comorbidities, including hypertension, DM, HF, CVD, and CKD. The prevalence of atrial fibrillation and stroke was high in the moderate burden group but not in the high burden group compared to the low burden group. In terms of the medication history, antiplatelet medications, angiotensin-converting enzyme inhibitor/angiotensin receptor blocker (ACEI/ARBs), and statins were more frequent in the moderate and high burden group. Regarding AADs, the use of class Ib AAD was more frequent in comparison with other type AADs for patients with a high VPC burden. The outcome before the propensity score matching analysis in the three groups showed higher all-cause mortality (VPC burden: low, 7.1%; moderate, 11.0%; and high, 10.2%) and cardiovascular mortality (VPC burden: low, 1.3%; moderate, 3.3%; and high, 3.7%) in the moderate and high burden groups in comparison with the low burden group (Table 1).

**Table 1:** Baseline characteristics, medication history, Holter data, and clinical outcome based on different VPC burdens.

	**Low burden (*N* = 16996)**	**Moderate burden (*N* = 1856)**	**High burden (*N* = 675)**	***P* value**
	**Median (IQR)/*N* (%)/Mean ±SD**	**Median (IQR)/*N* (%)/Mean ±SD**	**Median (IQR)/*N* (%)/Mean ±SD**	
**Clinical characteristics**				
Age (y)	59.2 ± 17.8	62.9 ± 16.1	60.9 ±16.4	<0.001
Male	7,793 (45.9)	1,048 (56.5)	380 (56.3)	<0.001
Follow-up days	987 (552–1428)	973 (527–1436)	963 (541–1441)	0.901
HTN	8,230 (48.4)	1038 (55.9)	352 (52.2)	<0.001
DM	3,653 (21.5)	520 (28.0)	194 (28.7)	<0.001
Dyslipidemia	7,679 (45.2)	972 (52.4)	330 (48.9)	<0.001
HF	1,824 (10.7)	440 (23.7)	160 (23.7)	<0.001
CVD	3,640 (21.4)	632 (34.1)	191 (28.2)	<0.001
CAD	1,841 (10.8)	374 (20.2)	120 (17.8)	<0.001
Stroke	1,437 (8.5)	206 (11.1)	54 (8.0)	0.001
PAD	362 (2.1)	52 (2.8)	17 (2.5)	0.020
AF	2,899 (17.1)	477 (25.7)	121 (17.9)	<0.001
CKD	3,197 (18.8)	499 (26.9)	170 (25.2)	<0.001
**Medication**				
Aspirin	3,461 (20.4)	485 (26.1)	146 (21.6)	<0.001
P_2_Y_12_ inhibitor	1,350 (7.9)	244 (13.2)	82 (12.2)	<0.001
ACEi/ARB	2,697 (15.9)	455 (24.5)	156 (23.1)	<0.001
Statin	3,059 (18.0)	414 (22.3)	139 (20.6)	<0.001
Class Ia AAD	7 (0.04)	1 (0.05)	0 (0)	0.839
Class Ib AAD	367 (2.2)	372 (21.0)	254 (39.6)	<0.001
Class Ic AAD	317 (1.9)	42 (2.3)	17 (2.5)	0.259
Class III AAD	903 (5.3)	236 (12.7)	117 (17.3)	<0.001
Beta blocker	3,478 (20.5)	484 (26.1)	161 (23.9)	<0.001
Non-DHP CCB	873 (5.1)	112 (6.0)	61 (9.0)	<0.001
**Holter data**				
VPC count	5 (1–44)	2,712 (1,618–4,982)	17,726 (13,306–25,704)	<0.001
VPC burden (%)	<0.1 (<0.1– <0.1)	2.6 (1.5–4.9)	17.5 (13.1–25.4)	<0.001
VT	544 (3.2)	418 (22.5)	263 (38.9)	<0.001
APC count	17 (3–104)	32 (3–566)	15 (2–107)	<0.001
Clinical outcome				
All-cause death	1,198 (7.1)	204 (11.0)	69 (10.2)	<0.001
CV death	214 (1.3)	61 (3.3)	25 (3.7)	<0.001

*AAD, antiarrhythmic drug; ACEI/ARB, angiotensin-converting enzyme inhibitor/angiotensin receptor blocker; AF, atrial fibrillation; APC, atrial premature complex; CKD, chronic kidney disease; CV death, cardiovascular death; CVD, cardiovascular disease (including coronary artery disease, stroke, and peripheral artery disease); DM, diabetes mellitus; HF, heart failure; HTN, hypertension; IQR, interquartile range; non-DHP CCB, non-dihydropyridine calcium channel blocker; VPC, ventricular premature complex. VT, ventricular tachycardia. Age was shown as mean ± SD. Follow-up days, VPC count, VPC burden, and APC count were shown as median (IQR). The other variables were shown as n (%)*.

To validate the effect of VPC burden on all-cause death and cardiovascular death, the daily VPC count was translated to logarithm due to a right skew deviation of the raw VPC daily count, which showed that the VPC burden (log VPC) was significantly higher in the all-cause death population (3.9 ± 3.0 vs. 2.9 ± 2.9, *p* < 0.001) and cardiovascular death (5.0 ± 3.0 vs. 2.9 ± 2.9, *p* < 0.001) population in comparison with the survival population in this cohort (Supplementary Tables 1, 2). Moreover, the daily burden of the cardiovascular death population was even higher than that of the all-cause death population. To further analyze the effect of the daily VPC burden on cardiovascular death, a Cox regression analysis and a competing risks survival analysis using the Fine–Gray subdistribution hazard model were performed (Figure 3 and Supplementary Table 3). In comparison with the low burden group, the moderate burden group showed a higher risk of cardiovascular death (multivariable Cox regression model adjusted hazard ratio (HR) 1.47, 1.09–1.98, *p* < 0.05, Fine and Gray's competing risk model adjusted HR 1.48, 1.09–2.01, *p* < 0.05). The trend was even higher in the high burden group (Cox regression model adjusted HR 1.68, 1.07–2.63, *p* < 0.05, Fine and Gray's competing risk model adjusted HR 1.70, 1.06–2.71 *p* < 0.05). These results indicated a moderate and high VPC burden led to more cardiovascular death. Regarding the all-cause death, neither the moderate burden group (multivariable Cox regression model adjusted HR 1.14, 0.98–1.33, *p* = 0.11) nor the high burden group (multivariable Cox regression model adjusted HR 1.10, 0.85–1.42, *p* = 0.16) showed a significantly higher risk in comparison with the low burden group.

### High Burden of VPC Led to More Cardiovascular Death Confirmed by Propensity Score Match Analysis and Kaplan–Meier Analysis

**Figure 2:**
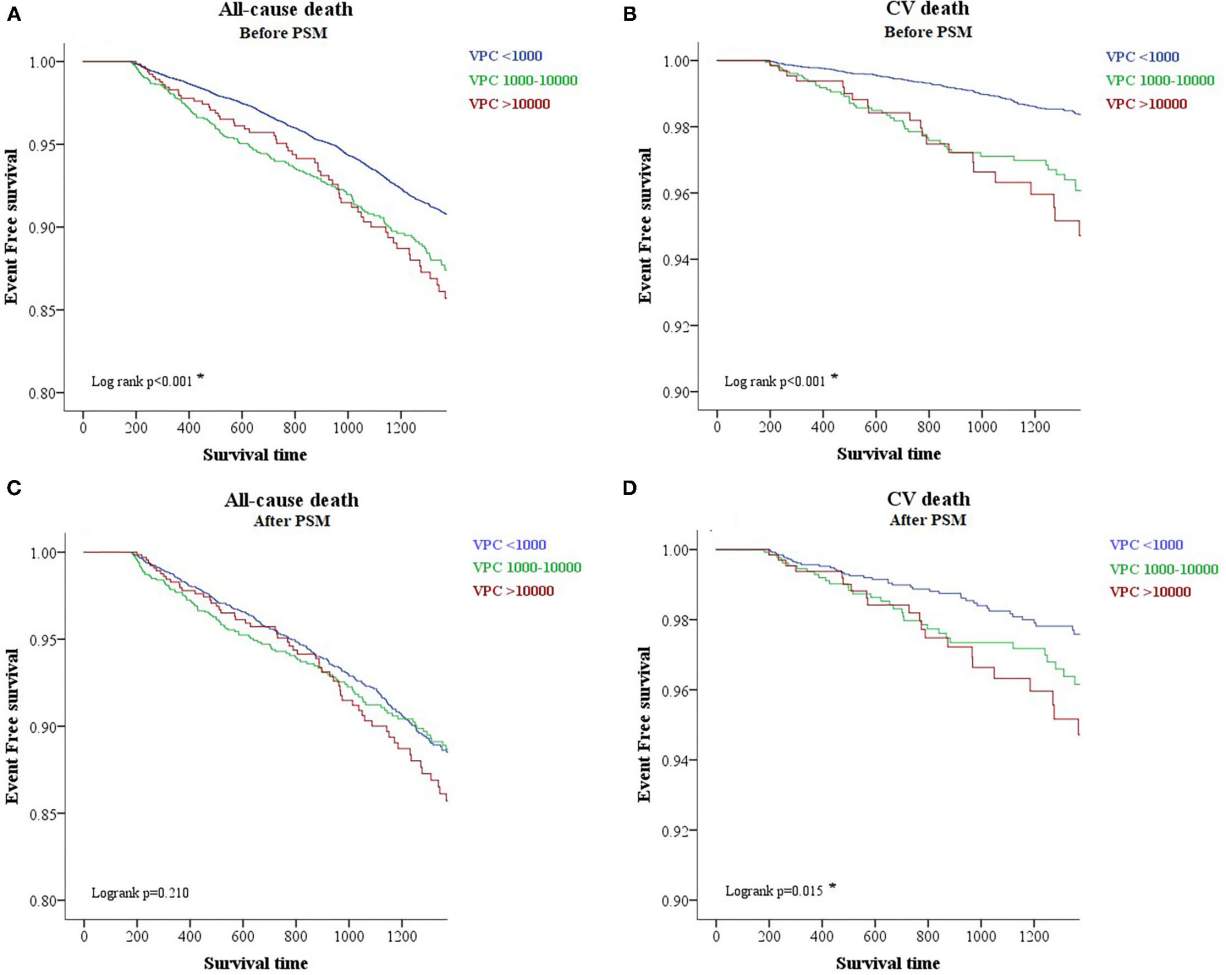
A Kaplan-Meier survival plot of different burdens of VPC regarding all-cause and cardiovascular death before and after the propensity score matching analysis. A Kaplan-Meier survival plot before the propensity score match analysis shows that a higher burden of VPC is associated with more all-cause and cardiovascular death **(A,B)**. After the propensity score matching analysis, however, the effect of a higher burden VPC leads to more cardiovascular death but not all-cause death **(C,D)**. CV death, cardiovascular death; PSM, propensity score match analysis.

To evaluate the prognostic effect of VPC burden on all-cause death and cardiovascular death, we tried to use the Kaplan–Meier analysis. The analysis demonstrated that a high VPC burden led to more all-cause death (log rank *p* < 0.001) and cardiovascular death (log rank *p* < 0.001) in comparison with a low burden (Figures 2A,B). However, clinical preexisting diseases had an impact on the outcome of all-cause and cardiovascular deaths, including age, gender, hypertension, DM, stroke, CKD, HF, and CVD. To elucidate whether a high and moderate VPC burden led to all-cause and cardiovascular mortality, a propensity score matching method was applied in this cohort study. Three individual groups were divided by the previous definitions and propensity score matched with a 4:2:1 ratio (Table 2). All-cause mortality was not different among the three groups (low, 9.0%; moderate, 10.6%; and high, 10.2%, *p* = 0.2129); however, both moderate and high VPC burdens led to more cardiovascular death in comparison with a low VPC burden (low, 2.1%; moderate, 3.3%; and high, 3.7%, *p* = 0.0125) (Table 2). The Kaplan–Meier analysis after a propensity score matching analysis also demonstrated the same trend. All-cause death did not show a statistical significance in these three groups (log rank *p* = 0.210, Figure 2C). On the other hand, the moderate and high burden groups showed a significant increase in cardiovascular death in comparison to the low burden group (log rank *p* = 0.015, Figure 2D). Based on the above statistical analyses, a moderate and high VPC burden led to more cardiovascular death compared with a low VPC burden.

**Table 2:** Clinical outcome after the propensity score matched group analysis for a low, moderate, and high burden of VPC (4:2:1 match).

	**Low (*N* = 2,700)**	**Moderate (*N* = 1,350)**	**High (*N* = 675)**	***P* value**
	**Mean (SD)/*N* (%)**	**Mean (SD)/*N* (%)**	**Mean (SD)/*N* (%)**	
Age	59.9 (16.7)	61.1 (16.5)	60.9 (16.4)	0.090
Male	1,492 (55.3)	754 (55.9)	380 (56.3)	0.864
DM	753 (27.9)	389 (28.8)	194 (28.7)	0.793
HTN	1,379 (51.1)	707 (52.4)	352 (52.2)	0.704
CKD	648 (24.0)	341 (25.3)	170 (25.2)	0.621
HF	660 (24.4)	315 (23.3)	160 (23.7)	0.722
CVD	499 (18.5)	248 (18.4)	127 (18.8)	0.970
All-cause death	242 (9.0)	143 (10.6)	69 (10.2)	0.213
CV death	56 (2.1)	45 (3.3)	25 (3.7)	**0.013**

*CKD, chronic kidney disease; CV death, cardiovascular death; CVD, cardiovascular disease (including coronary artery disease, stroke, and peripheral artery disease); DM, diabetes mellitus; HF, heart failure; HTN, hypertension; VPC, ventricular premature complex. Age was shown as mean (SD), and the other variables were shown as n (%). Bold value means statistically significant (p <0.05)*.

### Subgroup Analysis of Different Burdens of VPC and Cardiovascular Death

The subgroup analysis of the adjusted HRs for cardiovascular death compared the high burden group or moderate burden group with the low burden group (Figure 4). In terms of baseline characteristics, elderly patients (≥60 years old) with an underlying history of HF and hypertension, and patients who did not use antiplatelet drugs, beta blockers, classes Ib and III AADs, or non-dihydropyridine calcium channel blockers (non-DHP CCB) had a higher risk of cardiovascular death in the high burden group. In the moderate burden group, a similar trend was also observed. We conclude that elderly patients with underlying diseases, and those who do not use AADs, had a higher risk of cardiovascular death with moderate or high VPC burdens.

**Figure 3:**
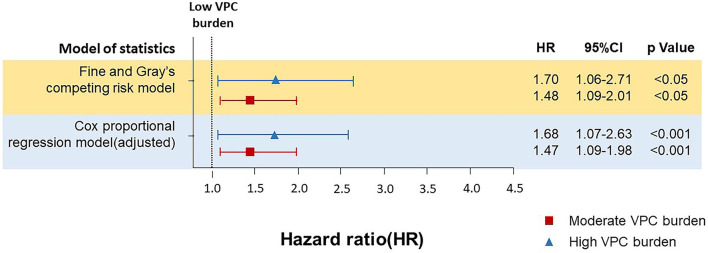
The Cox proportional regression model and Fine and Gray's competing risk model for analyzing the effect of different burdens of VPC on cardiovascular death. Both Cox proportional regression model and Fine and Gray's competing risk model demonstrated that in comparison with the low burden group, the moderate burden group showed a higher risk of cardiovascular death. The Cox proportional regression model and the Fine and Gray's competing risk model was adjusted with baseline characteristics and underlying diseases, including age, gender, diabetes mellitus (DM), dyslipidemia, hypertension, chronic kidney disease (CKD), heart failure (HF), cardiovascular diseases (CVDs), including coronary artery disease, stroke, and peripheral artery disease), ventricular tachycardia (VT), the use of antiarrhythmic drugs (AAD), and beta blocker.

**Figure 4:**
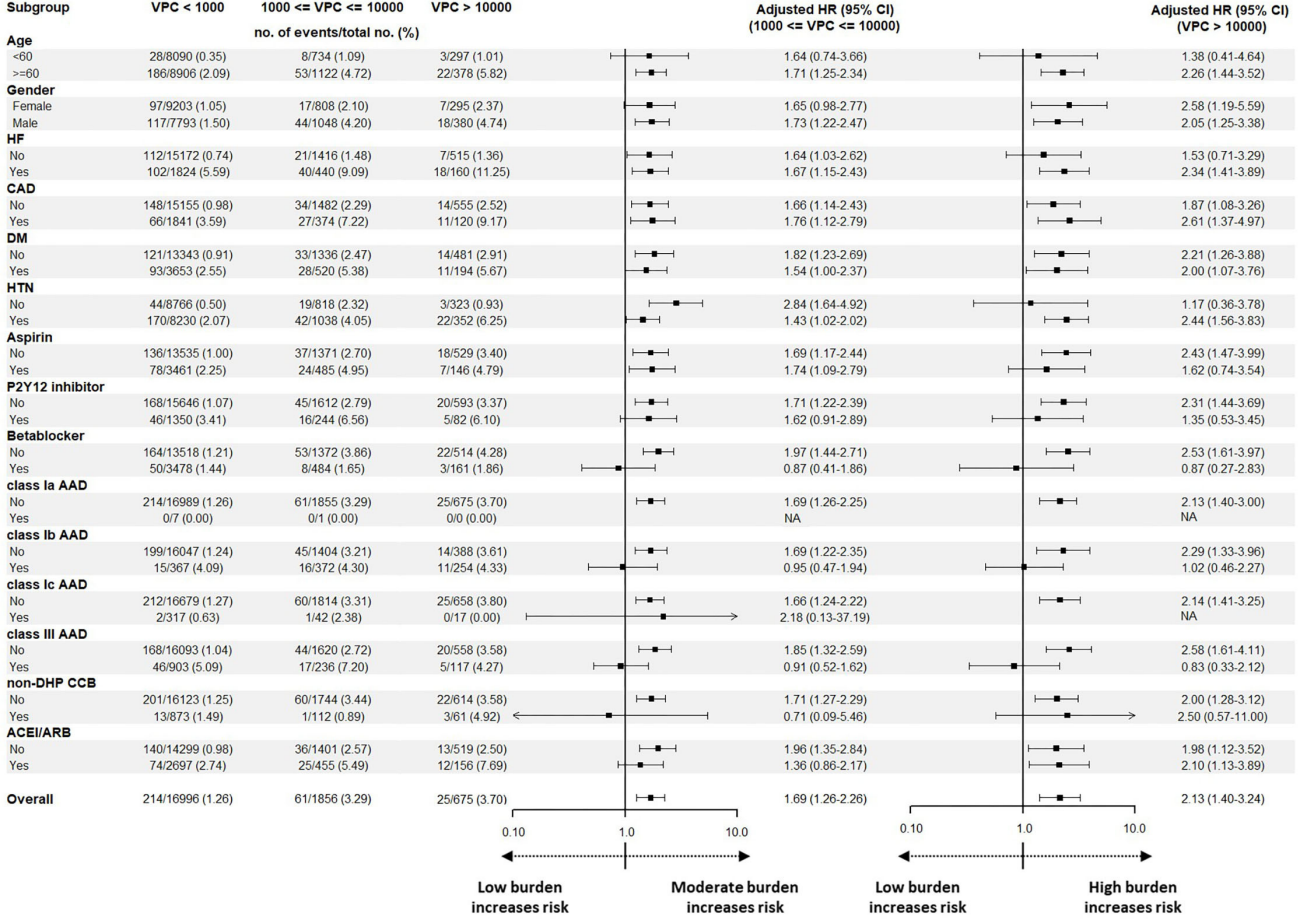
A subgroup analysis before the propensity match analysis for analyzing the effect of baseline characteristics, underlying disease, and medication on cardiovascular death. Illustration of the subgroup analysis before the propensity score match analysis. Male, elderly, patients without hypertension or DM, and patients who did not use P2Y12 inhibitor, beta blocker, non-DHP CCB, ACEI/ARB, or AADs showed an increased risk to have cardiovascular death in comparison with the low burden group. Regarding the high burden group, the elderly, patients with HF and hypertension, and patients who did not use aspirin, beta blockers, non-DHP CCB, or AADs showed an increased risk to have cardiovascular death in comparison to the low burden group. AAD, antiarrhythmic drug; ACEI/ARB, angiotensin-converting enzyme inhibitor/angiotensin receptor blocker; CAD, coronary artery disease; CI, confidence interval; DM, diabetes mellitus; HF, heart failure; HTN, hypertension; non-DHP CCB, non-dihydropyridine calcium channel blocker.

## Discussion

In a population-based cohort study of 19,527 patients who underwent 24-h Holter ECG monitoring in Taiwan, a high and moderate VPC burden was associated with a high risk of cardiovascular death. Moreover, in the high and moderate burden groups, patients without the use of cardioprotective drug s and AADs had higher risks of cardiovascular death.

### VPC and Cardiovascular Mortality

Previous cohort studies of VPC have shown that the frequent VPC was associated with increased all-cause mortality. In a previous study, which contained 5,778 patients with a mean follow-up period of 10 years, Lin et al. postulated that a cut-off value of 12 VPCs per day was predictive of all-cause mortality with an area under the receiver operating characteristics (ROC) curve of 59.6% (1). Another community-based study, which enrolled 15,792 patients, also demonstrated that the presence of VPC had a higher rate of subsequent HF as compared to no VPC on a 2-min rhythm ECG strip over an average follow-up of 15.6 years (20). However, the abovementioned two studies did not mention cardiovascular mortality. A recent subgroup analysis from the CHF-STAT study involving 421 patients reported a group of VPC-cardiomyopathy with a median count of 2,832 VPCs per day, which was associated with more cardiac death and resuscitated cardiac arrest (10). In our study, we collected 19,527 patients with continuous electrogram monitoring (Holter), and we also support that VPC burden of more than 1,000/day was associated with more cardiovascular death, which was consolidated by the propensity score match analysis. Furthermore, the relationship between VPC and cardiovascular death was tested by the univariate and multivariate Cox proportional hazards model and the Fine–Gray subdistribution hazard model (19, 21–23). In the field of cardiovascular research regarding disease incidence and prognosis prediction, the performance of the Fine–Gray subdistribution hazard model prevented overestimating the incidence of the outcome of interest when the competing risk was present (19). All these models yielded similar results. The abovementioned finding supports the hypothesis that VPC burden had a negative impact on cardiovascular mortality.

### Possible Mechanisms of VPC-Induced Cardiovascular Mortality

Besides the cohort studies, we postulated several possible mechanisms of VPC-induced cardiovascular death. First, a high VPC burden is associated with cardiomyopathy. The effect of premature contraction on the cardiac structural change was shown to be limited to the VPC, and the same situation did not appear in the atrial premature contraction (24). To demonstrate the ventricular structural change induced by VPC, a study of programmed pacemaker stimulation simulating persistent VPC in the canine models can induce functional changes in the myocardium and lead to a significant reduction of the left ventricular systolic function (25). Previous animal models also showed that left ventricular dys-synchrony and eccentric hypertrophy caused by VPC are also considered to be associated with the deterioration of the left ventricular systolic function (26, 27). Another swine model of VPC-induced cardiomyopathy model further demonstrated persistent changes in myocardial fibrosis and left ventricular dys-synchrony after the elimination of VPC (28). Left ventricular fibrosis and dys-synchrony caused by VPC were proven to be associated with the worse cardiovascular outcome (29, 30). The above studies provided insights that VPC made a significant impact on left ventricular functional and structural dysfunction and potentially led to more cardiac death. Second, electrical remodeling also has an important role. An animal and a cellular study proposed that the myocardial cells extracted from a chronic frequent VPC canine model can induce a heterogeneous reduction in Ito, IK1, and ICaL. The increased repolarization heterogeneity due to decreased outward and inward L-type calcium currents were associated with significant beat-to-beat action potential duration dispersion and may result in an increased risk of reentrant ventricular tachyarrhythmia, which is related to sudden cardiac death (31). Third, VPC can also induce cardiac autonomic dysregulation. VPC delivery in both human and porcine models elicited a significant cardiac sympathetic response than other stimuli (32, 33). In addition, VPC-induced sympathetic neural hyperactivity persisted despite the withdrawal of electrical stimulation and normalization of left ventricular ejection fraction (34). Cardiac autonomic dysregulation is an important trigger and substrate for ventricular pro-arrhythmia, leading to cardiac mortality.

### Risk Stratification of VPC in Cardiovascular Death

Recently, Voskoboinik et al. postulated an “ABC-VT” scoring system to evaluate the adverse outcome in a frequent VPC cohort including 206 patients. The ABC-VT score includes a superiorly directed VPC axis, a high VPC burden of 10–20% and >20%, a VPC coupling interval >500 ms, and the presence of non-sustained VT. Patients with frequent VPC and ABC-VT score >4 had a higher risk to have composite endpoints of a left ventricular ejection fraction decline by 10%, HF hospitalization, or cardiovascular mortality. In this study, the VPC burden of 10–20% and >20% had an odds ratio of 3.5 and 4.4, respectively, to meet the composite endpoints (35). In terms of our current study, the most significant advantage was the number of patients. Regarding the number of patients with a high VPC burden, we have 650 patients, which was three times more than the previous study. Moreover, the cohort study was large enough to arrange a comparison between a moderate VPC burden and a low VPC burden with a 1:2:4 ratio of propensity score match analysis. Our study demonstrated that not only the high VPC burden group but also the moderate VPC burden group experienced a higher risk of cardiovascular mortality than the low VPC burden group (3.7 vs. 3.3 vs. 2.1%, respectively; *p* = 0.0125) after the propensity score match analysis. Currently, there was no study that evaluated the effect of moderate VPC burden on long-term prognosis. In our opinion, medical treatment should be taken into consideration for patients with a moderate VPC burden who still had a higher risk of cardiovascular death compared with the low VPC burden group.

### Possible Risk Factors of High Burden VPC Associated With Cardiovascular Death

Heart failure patients with a high VPC burden were reported to be at a higher risk of cardiovascular events (36). In the subgroup analysis of the current study, not only HF but also hypertension and coronary artery disease contributed to a higher risk of cardiovascular death in the high VPC burden group. Furthermore, according to a previous study, the treatment to VPC was able to effectively reduce the VPC burden and prevent the decline of the left ventricular systolic function (37). In the subgroup analysis of our study, the use of cardioprotective drugs, including antiplatelet drugs, beta blockers, AADs, and non-DHP CCB, in the high VPC burden group is associated with less cardiovascular death. Although class Ic AADs were reported to be effective in the treatment of VPC-induced cardiomyopathy, the effect of other AADs, including mexiletine and amiodarone, beta blockers, and non-DHP CCB, were not reported to reduce cardiovascular death in high VPC burden patients in the literature (9). Comprehensive prospective studies were needed to clarify whether these drugs were effective for preventing cardiovascular death in patients with a high VPC burden.

### Limitations of This Study

Several limitations should be mentioned. First, this was a single-hospital, retrospective study based on a chart review. Although incomplete chart records or the loss of follow-up had been excluded from the study, the impact of the excluded cases on outcomes is unknown. Second, the study cohort was selected from a single center in Southern Taiwan. The populations included in the current study were mostly Asians. Whether our results could be applied to other races remained unknown. Third, only patients who were followed up more than 180 days were enrolled in the study. However, the median follow-up period of the study was only 955 days, which may not be long enough to trace the cardiovascular death although a competing risk analysis model was used to fix the disadvantage. Fourth, serial echocardiography data were not available during data mining, as a result, the VPC-induced cardiomyopathy or VPC-induced HF cannot be defined clearly and should not be mentioned in the current study. Finally, interventional data, including coronary intervention and catheter ablation, were not available during data mining. The effect of cardiovascular intervention to reduce mortality remained unknown.

## Conclusion

**Figure 5:**
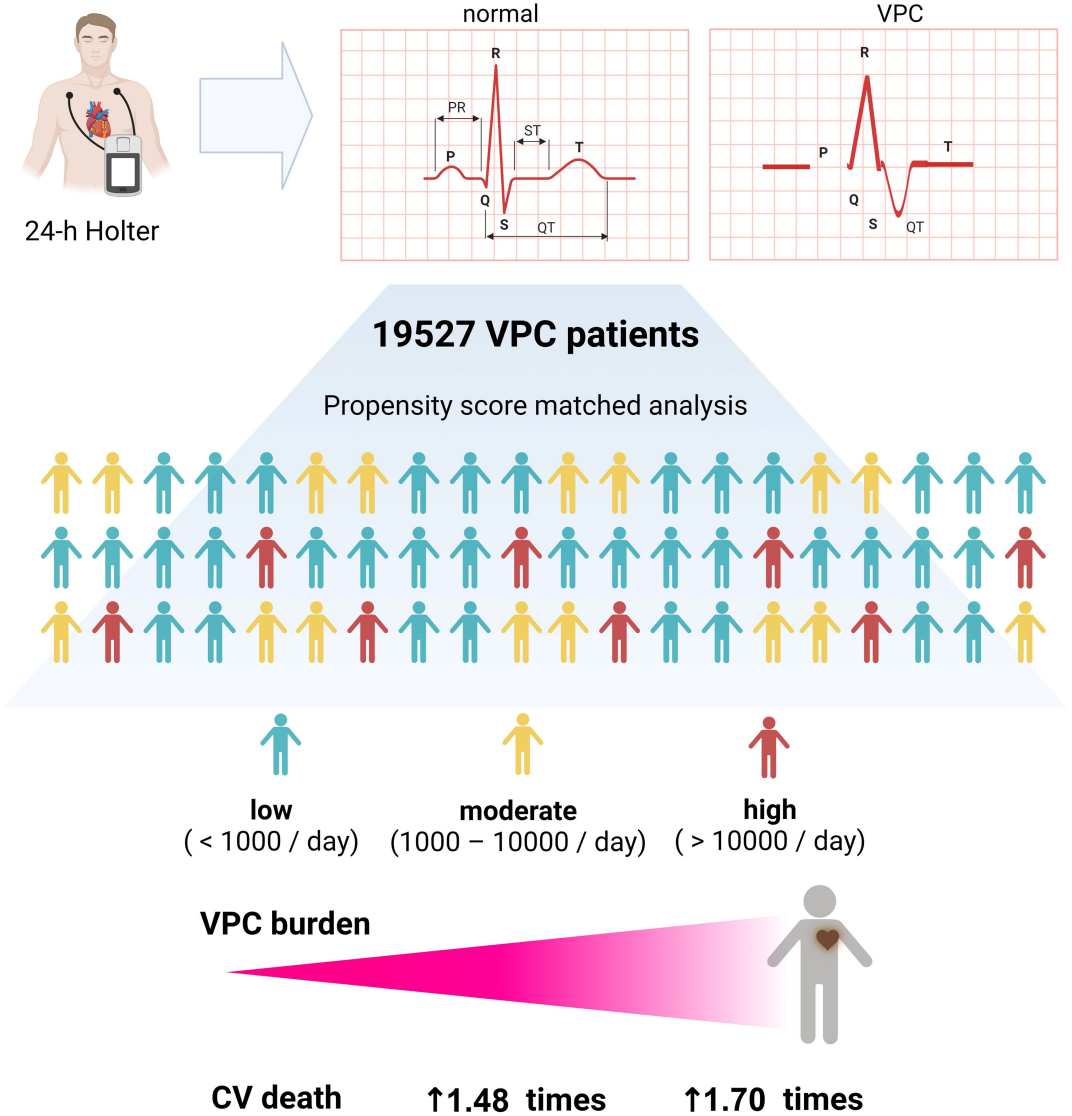
Graphic abstract of this study. A high and moderate VPC burden led to more cardiovascular death, and a high VPC burden had 1.70 times of cardiovascular death risk.

A higher VPC burden, particularly those with a high (>10,000/day) or moderate (1,000–10,000/day) burden, is an independent risk factor of cardiovascular mortality. Patients in the high VPC burden group were at a higher risk of experiencing cardiovascular death (Figure 5).

## Data Availability Statement

The data analyzed in this study is subject to the following licenses/restrictions: The data reported in this study cannot be deposited in a public repository because it was from the NCKUH-EMR database, and it did not open to the public. Requests to access these datasets should be directed to Ping-Yen Liu, larry@mail.ncku.edu.tw.

## Ethics Statement

The studies involving human participants were reviewed and approved by National Cheng Kung University Hospital. Written informed consent for participation was not required for this study in accordance with the national legislation and the institutional requirements.

## Author Contributions

P-TL and T-CH: conceptualization. M-HHua: data curation. M-HHua, P-FS, and M-HHun: formal analysis and methodology. P-YL: funding acquisition and supervision. P-TL and P-YL: investigation. P-TL, T-CH, M-HHua, Y-WL, and P-YL: project administration. M-HHua and M-HHun: software. P-TL: writing—original draft. L-WH and P-YL: writing—review and editing. All authors contributed to the article and approved the submitted version.

## Funding

This study was funded by National Cheng Kung University Hospital, Tainan, Taiwan (Nos. NCKUH-11104046 and NCKUH-10902023), National Cheng-Kung University for its grant (Nos. MOST-109-2634-F-006-023 and MOST-110-2634-F-006-020), and Ministry of Science and Technology of Taiwan.

## Conflict of Interest

The authors declare that the research was conducted in the absence of any commercial or financial relationships that could be construed as a potential conflict of interest.

## Publisher's Note

All claims expressed in this article are solely those of the authors and do not necessarily represent those of their affiliated organizations, or those of the publisher, the editors and the reviewers. Any product that may be evaluated in this article, or claim that may be made by its manufacturer, is not guaranteed or endorsed by the publisher.
