# Supplementary material for: The Burden of Ventricular Premature Complex Is Associated With Cardiovascular Mortality
Source: Front Cardiovasc Med. 2022 Feb 3;8:797976. doi: 10.3389/fcvm.2021.797976 (PMC8850345; doi:10.3389/fcvm.2021.797976)
Supplement: Supplementary file 1 [file Data_Sheet_1.pdf]

Supplementary Material

1.1 Supplementary Tables

Table S1. Demographic and clinical characteristics of overall study cohort, event group, and event-free group regarding to all-cause mortality

	**Overall (N = 19527)**	**Event group (N = 1471)**	**Event-free group (N = 18056)**	***P* value**
	**Median (IQR) / N (%) / Mean±SD**	**Median (IQR) / N (%) / Mean±SD**	**Median (IQR) / N (%) / Mean±SD**	
Age (y)	59.6 ± 17.5	73.1 ± 12.5	58.5 ± 17.4	**< .001**
Male	9221 (47.2)	865 (58.8)	8356 (46.3)	**< .001**
Follow-up days	985 (549 – 1429)	860 (483 – 1255)	995.6 (555 – 1433)	**< .001**
APC	18.0 (3.0 – 117.0)	77.0 (9.0 – 654.0)	16.0 (3.0 – 98.0)	**< .001**
VPC	8.0 (1.0 – 155.0)	37.0 (3.0 – 434.0)	8.0 (1.0 – 138.0)	**< .001**
HTN	9620 (49.3)	1056 (71.8)	8564 (47.4)	**< .001**
DM	4367 (22.4)	607 (41.3)	3760 (20.8)	**< .001**
Dyslipidemia	8981 (46.0)	706 (48.0)	8275 (45.8)	0.115
HF	2424 (12.4)	481 (32.7)	1943 (10.8)	**< .001**
AMI	749 (3.8)	116 (7.9)	633 (3.5)	**< .001**
CAD	2335 (12.0)	333 (22.6)	2002 (11.1)	**< .001**
PAD	431 (2.2)	80 (5.4)	351 (1.9)	**< .001**
AF	3497 (17.9)	544 (37.0)	2953 (16.4)	**< .001**
Stroke	1697 (8.7)	257 (17.5)	1440 (8.0)	**< .001**
CKD	3866 (19.8)	760 (51.7)	3106 (17.2)	**< .001**
CVD	2595 (13.3)	372 (25.3)	2223 (12.3)	**< .001**
Aspirin	4092 (21.0)	378 (25.7)	3714 (20.6)	**< .001**
P_2Y_{12} inhibitor	1676 (8.6)	217 (14.8)	1459 (8.1)	**< .001**
ACEi/ARB	3308 (16.9)	322 (21.9)	2986 (16.5)	**< .001**
Statin	3612 (18.5)	262 (17.8)	3350 (18.6)	0.503
Class Ia	8 (0.04)	3 (0.2)	5 (0.03)	**0.018**
Class Ib AAD	993 (5.3)	99 (6.7)	884 (5.2)	**0.011**
Class Ic AAD	376 (1.9)	22 (1.5)	354 (2.0)	0.250
Class III AAD	1256 (6.4)	202 (13.7)	1054 (5.8)	**< .001**
Beta blocker	4123 (21.1)	261 (17.7)	3862 (21.4)	**0.001**
Non-DHP CCB	1046 (5.4)	126 (8.6)	920 (5.1)	**< .001**

AAD, antiarrhythmic drug; ACEI/ARB, angiotensin-converting enzyme inhibitor/angiotensin receptor blocker; AF, atrial fibrillation; APC, atrial premature complex; AMI, acute myocardial infarction; CAD, coronary artery disease; CKD, chronic kidney disease; CV death, cardiovascular death ;CVD, cardiovascular disease (including coronary artery disease, stroke, and peripheral artery

disease); DM, diabetes mellitus; HF, heart failure; HTN, hypertension; IQR, interquartile range; non-DHP CCB, non-dihydropyridine calcium channel blocker; PAD, peripheral artery disease; VPC, ventricular premature complex. VT, ventricular tachycardia. Age was shown as Mean ± SD. Follow-up days, VPC count, VPC burden, APC count were shown as Median (IQR). The other variables were shown as N (%).

Table S2. Demographic and clinical characteristics of overall study cohort, event group, and event-free group regarding to cardiovascular death

	**Overall (N = 19527)**	**Event group (N = 300)**	**Event-free group (N = 19227)**	***P* value**
	**Median (IQR) / N (%) / Mean±SD**	**Median (IQR) / N (%) / Mean±SD**	**Median (IQR) / N (%) / Mean±SD**	
Age (y)	59.6 ± 17.5	73.3 ± 13.2	59.4 ± 17.5	**< .001**
Male	9221 (47.2)	179 (59.7)	9042 (47.0)	**< .001**
Follow-up days	985 (549 – 1429)	850 (479 – 1327)	987 (551 – 1429)	**0.002**
APC	18 (3 – 117)	58 (0 – 681)	18 (3 – 113)	**< .001**
VPC	8 (1 – 155)	227 (11 – 1401)	8 (1 – 146)	**< .001**
HTN	9620 (49.3)	234 (78.0)	9386 (48.8)	**< .001**
DM	4367 (22.4)	132 (44.0)	4235 (22.0)	**< .001**
Dyslipidemia	8981 (46.0)	175 (58.3)	8806 (45.8)	**< .001**
HF	2424 (12.4)	160 (53.3)	2264 (11.8)	**< .001**
AMI	749 (3.8)	47 (15.7)	702 (3.7)	**< .001**
CAD	2335 (12.0)	104 (34.7)	2231 (11.6)	**< .001**
PAD	431 (2.2)	24 (8.0)	407 (2.1)	**< .001**
AF	3497 (17.9)	152 (50.7)	3345 (17.4)	**< .001**
Stroke	1697 (8.7)	57 (19.0)	1640 (8.5)	**< .001**
CKD	3866 (19.8)	194 (64.7)	3672 (19.1)	**< .001**
CVD	2595 (13.3)	113 (37.7)	2482 (12.9)	**< .001**
Aspirin	4092 (21.0)	109 (36.3)	3983 (20.7)	**< .001**
P_2Y_{12} inhibitor	1676 (8.6)	67 (22.3)	1609 (8.4)	**< .001**
ACEi/ARB	3308 (16.9)	111 (37.0)	3197 (16.6)	**< .001**
Statin	3612 (18.5)	74 (24.7)	3538 (18.4)	**0.007**
Class Ia AAD	8 (0.04)	0 (0)	8 (0.04)	1.000
Class Ib AAD	993 (5.3)	42 (14.0)	951 (5.1)	**< .001**
Class Ic AAD	376 (1.9)	3 (1.0)	373 (1.9)	0.335
Class III AAD	1256 (6.4)	68 (22.7)	1188 (6.2)	**< .001**
Beta blocker	4123 (21.1)	61 (20.3)	4062 (21.1)	0.793
Non-DHP CCB	1046 (5.4)	17 (5.7)	1029 (5.4)	0.912

AAD, antiarrhythmic drug; ACEI/ARB, angiotensin-converting enzyme inhibitor/angiotensin receptor blocker; AF, atrial fibrillation; APC, atrial premature complex; AMI, acute myocardial infarction; CAD, coronary artery disease; CKD, chronic kidney disease; CV death, cardiovascular death; CVD, cardiovascular disease (including coronary artery disease, stroke, and peripheral artery disease); DM, diabetes mellitus; HF, heart failure; HTN, hypertension; IQR, interquartile range; non-DHP CCB, non-dihydropyridine calcium channel blocker; PAD, peripheral artery disease; VPC, ventricular premature complex. VT, ventricular tachycardia. Age was shown as Mean ± SD. Follow-

up days, VPC count, VPC burden, APC count were shown as Median (IQR). The other variables were shown as N (%).

Table S3. Univariate and multivariable Cox regression model for cardiovascular mortality.

	Crude HR (95% CI)	*P* value	Adjust HR (95% CI)	*P* value
VPC burden				
Low	Reference		Reference	
Moderate	2.60 (1.95-3.45)	<0.001	1.47 (1.09-1.98)	**0.012**
High	2.87 (1.89-4.34)	<0.001	1.68 (1.07-2.63)	**0.023**
Clinical characteristics				
Age	1.06 (1.05-1.07)	<0.001	1.04 (1.03-1.05)	**<0.001**
Male	1.67 (1.33-2.11)	<0.001	1.22 (0.96-1.54)	0.103
DM	2.63 (2.10-3.31)	<0.001	1.19 (0.93-1.53)	0.176
Dyslipidemia	1.55 (1.23-1.94)	<0.001	0.86 (0.67-1.11)	0.245
HTN	3.31 (2.52-4.35)	<0.001	1.06 (0.78-1.43)	0.716
CKD	7.19 (5.67-9.12)	<0.001	2.84 (2.18-3.70)	**<0.001**
HF	8.30 (6.61-10.41)	<0.001	3.89 (3.04-4.98)	**<0.001**
CVD	3.98 (3.15-5.03)	<0.001	1.57 (1.20-2.06)	**0.001**
VT count	1.001 (1.000-1.001)	0.003	1.00 (1.00-1.00)	0.989
Medication				
Antiplatelet	2.58 (2.06-3.24)	<0.001	1.10 (0.85-1.42)	0.470
AAD	3.29 (2.59-4.19)	<0.001	1.61 (1.24-2.09)	**<0.001**
Beta blocker	0.89 (0.67-1.18)	0.422	0.90 (0.68-1.20)	0.471

AAD, antiarrhythmic drug; CKD, chronic kidney disease; CVD, cardiovascular disease (including coronary artery disease, stroke, and peripheral artery disease); DM, diabetes mellitus; HF, heart failure; HTN, hypertension; IQR, interquartile range; non-DHP CCB, non-dihydropyridine calcium channel blocker; PAD, peripheral artery disease; VPC, ventricular premature complex. VT, ventricular tachycardia.
